# Treatment‐Free Remissions in Children With Chronic Myeloid Leukemia (CML): A Prospective Study From the Tata Memorial Hospital (TMH) Pediatric CML (pCML) Cohort

**DOI:** 10.1002/ajh.27528

**Published:** 2024-11-20

**Authors:** Nirmalya Roy Moulik, Swaminathan Keerthivasagam, Gaurav Chatterjee, Jayesh Agiwale, Pallavi Rane, Chetan Dhamne, Akanksha Chichra, Shyam Srinivasan, Purvi Mohanty, Hemani Jain, Dhanlaxmi Shetty, Sweta Rajpal, Prashant Tembhare, Nikhil Patkar, Gaurav Narula, Papagudi G. Subramanian, Shripad Banavali

**Affiliations:** ^1^ Tata Memorial Hospital Pediatric Oncology Mumbai India; ^2^ Homi Bhabha National Institute Mumbai India; ^3^ Jawaharlal Institute of Postgraduate Medical Education and Research, Medical Oncology Puducherry India; ^4^ Tata Memorial Hospital Hematopathology Mumbai India; ^5^ Tata Memorial Hospital Biostatistics Mumbai India; ^6^ Tata Memorial Hospital Cancer Cytogenetics Mumbai India

**Keywords:** imatinib discontinuation, pediatric CML, TFR in pediatric CML, TKI discontinuation

## Abstract

Pediatric chronic myeloid leukemia (pCML) is a rare childhood malignancy, representing 2%–3% of all childhood leukemia. Tyrosine kinase inhibitors (TKIs) have greatly improved survival but pose challenges due to their long‐term effects on growth and bone health in children. We prospectively studied treatment‐free remission (TFR) in 45 children with pCML in chronic phase on imatinib. Eligibility criteria were as per current NCCN guidelines, with a less stringent qPCR monitoring scheduled every 3 months. TFR was successful in 71.1% (32 out of 45) of patients after a median follow‐up of 25 (range: 6–42) months. The TFR rates at 12 and 24 months were 70% and 66%, respectively. Children under 5 years had a TFR rate of 88.9%, compared to 61.8% in those over 5 years (*p* = 0.18). Eleven of the 13 patients who lost MMR did so within 6 months of discontinuation. The cumulative incidence of loss in MMR at 6, 12, and 24 months was 26.4%, 27%, and 33%, respectively. Ten out of 13 (76.9%) patients with discontinuation failure (DF) regained MMR within 3 (2–20) months of restarting imatinib. A significant correlation was found between higher T‐regulatory cell levels at baseline and DF (*p* = 0.005). More than half patients showed improved bone mineral density after 2 years of TFR. Our findings suggest that high TFR rates can be attained in pCML, with added benefits for bone health. Less frequent molecular monitoring was not associated with adverse outcomes and there seems to be a role of the immune system in sustaining TFR. The study is registered in the Clinical Trials Registry‐India (CTRI/2020/11/029199).

## 1 Introduction

Chronic myeloid leukemia (CML) is a rare childhood malignancy, accounting for 2% to 3% of all leukemias in children [1]. The survival rate for CML patients has significantly improved to match that of healthy peers since the advent of tyrosine kinase inhibitors (TKIs) [2]. However, children face unique challenges compared to adults due to the off‐target effects of TKIs during the period of growth and organ maturation. The adverse impact of TKIs on growth in children is well‐documented [3, 4, 5]. TKIs like imatinib also affect bone remodeling and mineralization, leading to poor bone health in these children [6]. Additional TKI‐related toxicities include delayed puberty, infertility, and other endocrinopathies [5]. Furthermore, lifelong TKI treatment poses a significant financial burden and affects the quality of life for these children.

Adult studies on TKI discontinuation, such as STIM1 and EURO‐SKI, have demonstrated successful treatment‐free remission (TFR) in ~40% to 60% [7, 8] leading to substantial benefits in terms of quality of life and finances. Given the unique off‐target effects of TKIs in children, achieving TFR could provide substantial benefits during critical periods of growth and development. However, published data on TFR in pediatric chronic myeloid leukemia (pCML) is scarce. To date, only about 50–60 children have been reported in prospective and retrospective studies as having attempted treatment discontinuation [9, 10, 11, 12, 13]. We present our experience with the prospective discontinuation of imatinib in children with chronic myeloid leukemia in the chronic phase (CML‐CP) within a low‐resourced setting, marking the largest prospective pediatric TFR cohort reported so far.

## 2 Methods

We conducted a prospective study on discontinuation of imatinib in children diagnosed with CML‐chronic phase (CML‐CP) (less than 15 years of age at diagnosis) and treated at the Pediatric Oncology Department of Tata Memorial Hospital, Mumbai, India.

**FIGURE 1:**
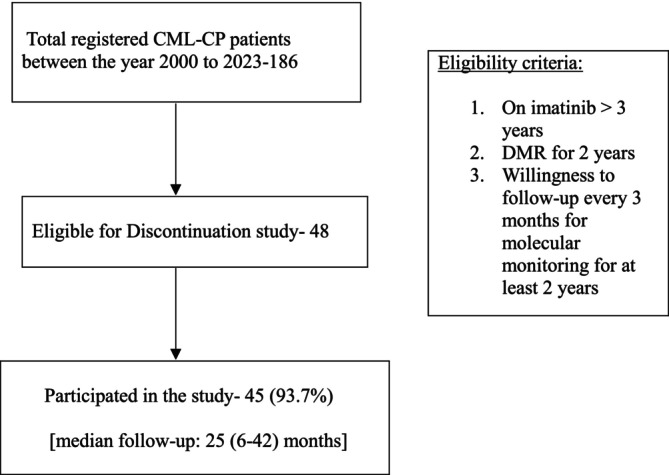
Consort diagram of the study.

Discontinuation of imatinib was offered to all such patients with CML‐CP satisfying the following eligibility criteria, adapted from NCCN stopping criteria for adults [14]: (a) on imatinib for more than 3 years, (b) persistent deep molecular response (DMR) with transcript levels less than 0.01% (MR4) for more than 2 years, and (c) willingness to come for frequent follow‐up visits for quantitative reverse transcription polymerase chain reaction (qPCR) testing. Although patients were advised to follow up for qPCR every 3 months, patients also had a monthly complete blood count for the first 3 months done locally and reviewed electronically. The consort diagram of the study is shown in Figure 1.

Baseline data, including the duration of imatinib treatment and BCR::ABL1 transcripts at different time points before discontinuation, were collected from case files and electronic hospital records. During follow‐up after discontinuation, patients with two consecutive rising transcript levels, repeated 4 weeks apart, over the level of major molecular response (MMR) (BCR::ABL1 transcripts > 0.1%) were considered as discontinuation failure (DF) and restarted on imatinib at the pre‐discontinuation dose. However, for patients with transcript levels beyond the level of 1%, imatinib was restarted without a repeat test. Following the resumption of imatinib, BCR::ABL1 transcript levels were monitored every 3 months till MMR (MR3), followed by response‐based frequency of monitoring.

The primary endpoint was the rate of TFR without molecular relapse at 6 months. Secondary endpoints included the evaluation of prognostic factors potentially associated with the persistence of molecular remission after imatinib discontinuation, such as age at diagnosis, duration of imatinib treatment, and duration of DMR until imatinib discontinuation. As an exploratory endpoint, absolute counts and relative proportions of 30 different T and NK cell subsets from peripheral blood, measured by flow cytometry at baseline and at each qPCR follow‐up until 1 year were studied and correlated with outcomes of the discontinuation trial.

## 3 Immune Cell (T and NK Cells) Characterization Using Flowcytometry

The flow cytometry processing, acquisition, and analysis strategy were modified from our previously published approach [15]. Briefly, fresh peripheral blood (PB) samples were processed by the stain–lyse–wash method using a custom‐designed 12‐color antibody panel (Table S1). PB samples were incubated with the 12‐color antibody cocktail at a pre‐titrated volume. A total of 100 000–300 000 cells were acquired on an LSR Fortessa flow cytometer (BD Biosciences). Data analysis was performed using a predesigned template‐based approach on Kaluza version 2.1 (Beckman Coulter, Inc.). Various immune cells (CD4+ and CD8+ T cells, γδ‐T cells, Tregs, NK cells, early, effector, and terminal NK cells, and CD159a + exhausted NK cells) were gated using previously published strategy [15, 16, 17, 18, 19] (Figure S1). A dual‐platform strategy was used to calculate absolute counts of various immune cells [20].

## 4 Statistics

Descriptive statistics were used for demographics and disease characteristics. Molecular relapse‐free survival was estimated using the Kaplan–Meier method. Cumulative incidence of loss in MMR post‐discontinuation and gain in MMR post‐reinitiation of imatinib were estimated using the cumulative incidence function, accounting for competing events. Associations between quantitative differences in various T/NK subsets at the time of discontinuation in groups with continued TFR and DF were evaluated using the Mann–Whitney *U*‐test or *t*‐test (depending on whether the normal distribution was absent or present), and cut‐off levels were determined using ROC curves. Associations between changes in levels of immune cells over time with DF or TFR groups were assessed using the Wilcoxon signed‐rank test. Statistical analysis was performed using R version 4.4.1 and Python version 3.12.4.

## 5 Results

Of the 186 children with CML‐CP enrolled in the pediatric oncology services of our hospital, 48 (25.8%) patients were eligible for discontinuation, and 45 of them agreed to participate in the discontinuation trial at the time of last analysis. The median (range) age of the 45 patients (Male: Female—3:1) at diagnosis of CML and imatinib discontinuation was 10 (1–14) and 20 (6–32) years, respectively. Nine out of 45 (20%) children were diagnosed at an age of less than 5 years. None of the patients had undergone allogeneic stem cell transplant before discontinuation. The median (range) duration of imatinib therapy was 126 (46–262) months. The median (range) time to attain MMR and DMR was 50  (7–155) months and 73 (10–235) months, respectively. The median (range) time for patients in sustained MMR and DMR was 75 (25–107) months and 54 (25–89), respectively.

**FIGURE 2:**
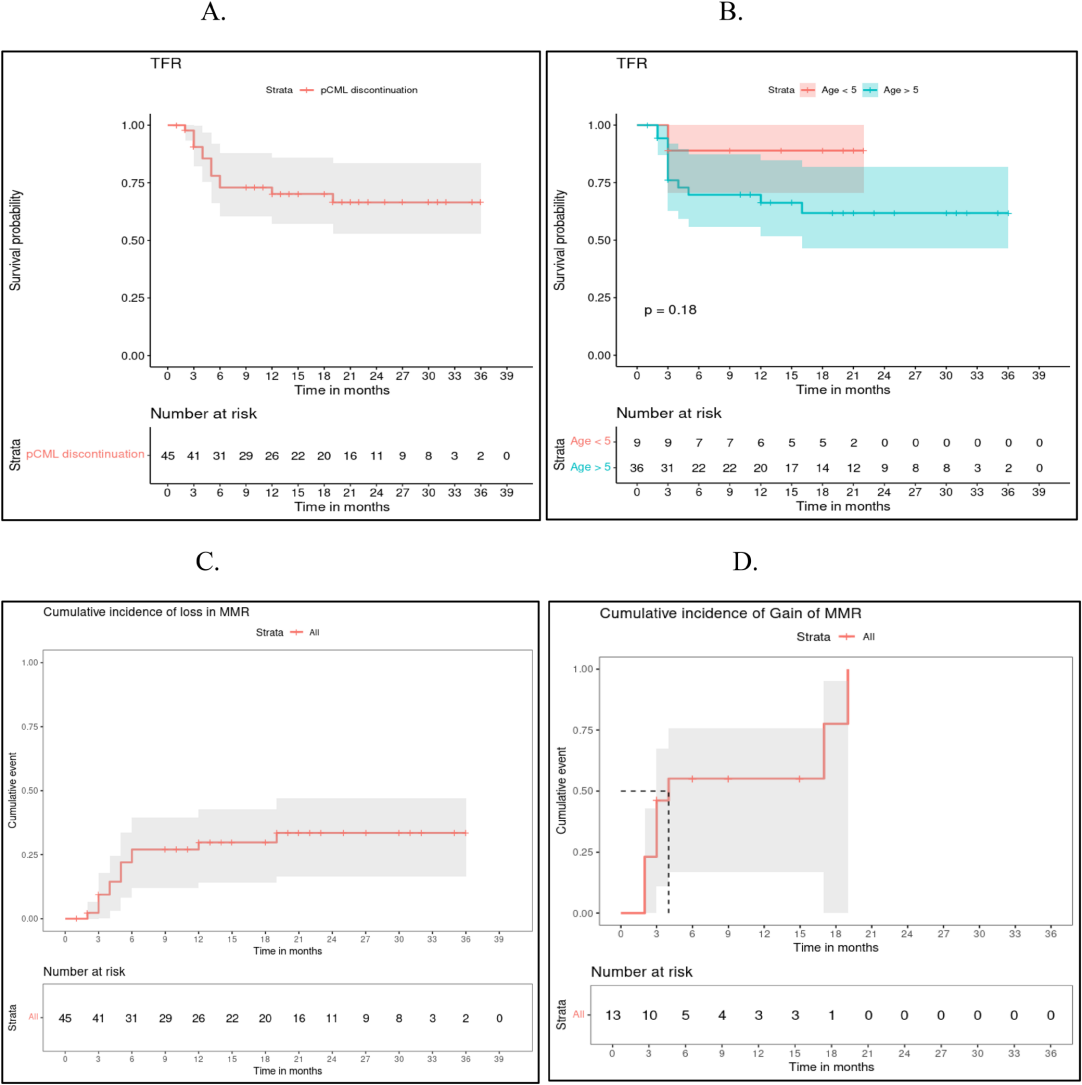
Kaplan Meier survival curves predicting (A). TFR rate in the entire cohort (B). TFR rate in children below and above 5 years at diagnosis (C). Cumulative incidence of loss of MMR post‐discontinuation of imatinib. (D) Cumulative incidence of gain of MMR after restart of imatinib. [Color figure can be viewed at wileyonlinelibrary.com]

Thirty‐two (71.1%) out of 45 patients had successful TFR and remained off imatinib after a median follow‐up of 25 months (range: 6–42 months). The TFR rates at 12 and 24 months were 70% (95% CI 61%–88%) and 66% (95% CI 52%–84%), respectively. The TFR rate for children below 5 and above 5 years at diagnosis was 88.9% and 61.8% (*p*‐value = 0.18), respectively (Figure 2A,B). Thirteen patients (28.9%) who experienced loss of MMR were restarted on imatinib. Eleven out of these 13 patients lost MMR within 6 months of imatinib discontinuation, while 2 patients did so after 6 months. The median (range) duration to loss of MMR after discontinuation was 3 (2–16) months. The cumulative incidence of loss in MMR at 6, 12, and 24 months was 26.4% (11.7–38.7), 27% (95% CI 12%–39%), and 33% (95% CI 16%–46%), respectively (Figure 2C). Imatinib was restarted at the pre‐discontinuation dose, and 10 out of the 13 patients (76.9%) with DF regained MMR at a median (range) duration of 3 (2–20) months. The cumulative incidence of regaining MMR at 12 and 24 months was 63% (95% CI 28%–84%) and 87% (95% CI 20%–98%), respectively (Figure 2D). Higher transcript levels (> 1%) at the time of molecular relapse post‐discontinuation were associated with a longer mean (SD) time to regain MMR (6.7[6.5] vs. 2[0] months, [*p* = 0.01]). There was no significant association between age, gender, duration of DMR, time to attain DMR (*p* = 0.168), MMR (*p* = 0.662), and successful TFR. Although children who were < 5 years of age at diagnosis had a higher TFR rate than their older counterparts, this was not statistically significant (Figure 2B).

**FIGURE 3:**
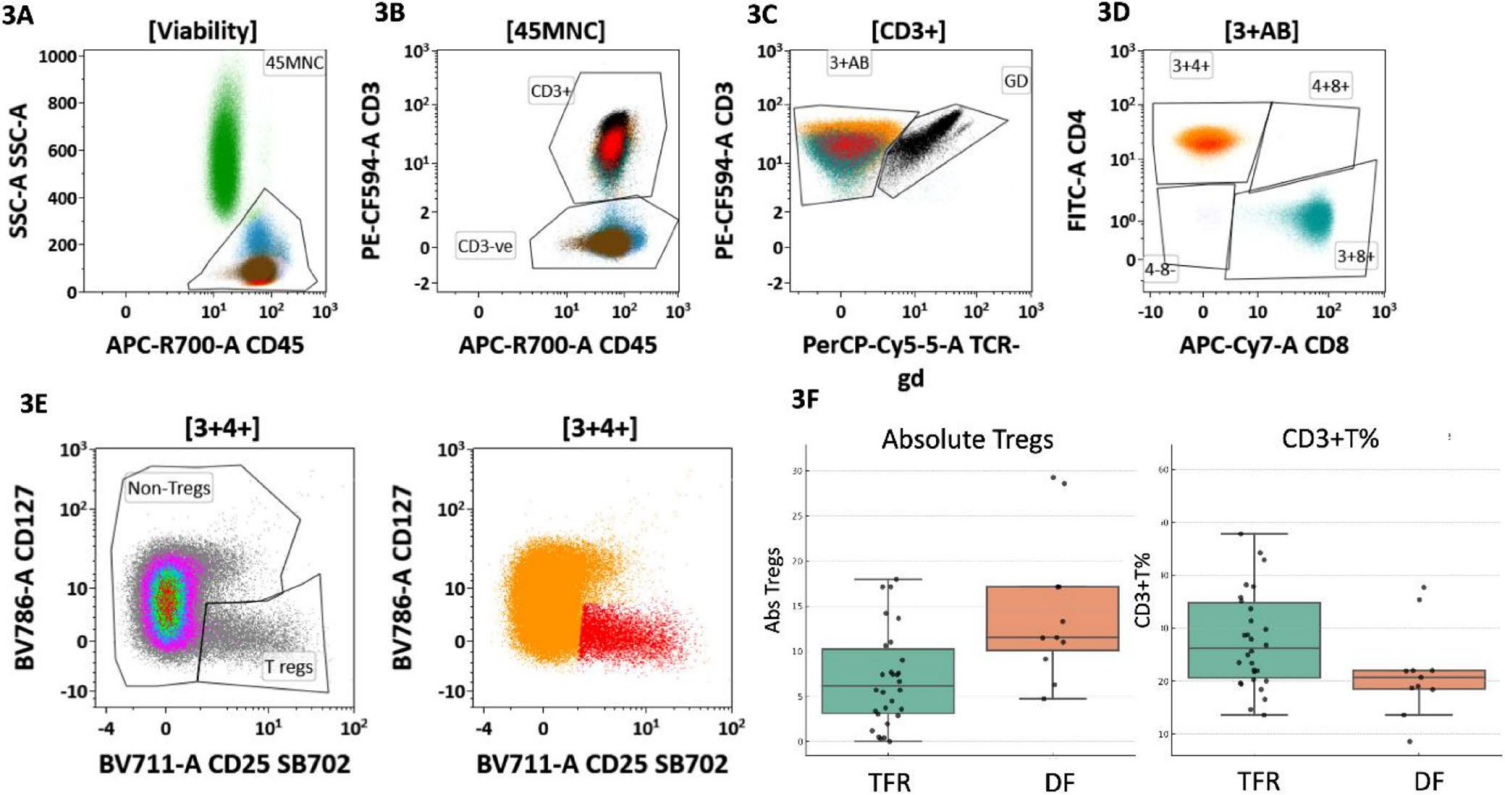
(A–E) shows a sequential gating strategy to identify the Tregs. Figure 3F shows box and dot plots showing the distribution of absolute Tregs and % of CD3+ T cells across the DF and TFR groups. After identifying the CD45+ mononuclear cells (Figure 3A), the T cells are gated using CD3 (Figure 3B). This is followed by the identification of CD4+ non‐γδ T cells (Figure 3C,D). Finally, the Tregs are identified using CD25 bright CD127 low expression among CD4+ non‐γδ T cells (Figure 3E). [Color figure can be viewed at wileyonlinelibrary.com]

We further looked at the association of various immune cells and patterns of change every 3 monthly for the initial 12 months in these children. At baseline, a lower median percentage (out of all viable cells) of CD3+ T cells (21.6% [range: 8.6%–37.7%] vs. 28.5% [range: 13.6%–61.9%], *p* = 0.04) and a higher median Tregs levels (absolute Tregs 11.5/μL [range: 4.7–29.3/μL] vs. 6.2/μL [range: 0–31.3/μL], *p* = 0.007) was observed in DF group compared to the TFR group (Figure 3). No statistically significant differences were observed in the levels of baseline absolute NK (median 88.8/μL vs. 83.8/μL, *p* = 0.74), effector NK (median 75.7/μL vs. 79.6/μL, *p* = 0.76), or CD159a + exhausted NK cell levels (median 26.4/μL vs. 36.9/μL, *p* = 0.86) across the DF and TFR groups. ROC analysis further determined the most appropriate cut‐off levels for absolute Tregs as 9.14/μL (AUC 0.78. sensitivity 82%, specificity 73%) with a > 9.14/μL absolute Tregs level being significantly associated with DF (*p* = 0.0048) (Figure S2). On evaluating the change in the levels of immune cells 3 months after discontinuation, no significant differences in the pattern of change were observed among the DF and TFR groups. Among 17 (64.7%) children who were in continued TFR with baseline < 9.14/μL Tregs level, only 6 (35.3%) showed an increase in Tregs level to > 9.14/μL at 3 months (Figure S3).

Ten of the 32 patients continuing in the TFR group have lost DMR after discontinuation but continue to have transcript levels below MMR, the cut‐off to restart (Figure S4). In three of these patients, qPCR levels transiently rose beyond MMR after discontinuing imatinib but dropped below the level of DMR in the repeat test done a month later. T‐reg/NK cell levels were not associated with the transient rise in transcript levels in these patients.

Four out of 45 patients (8.8%) experienced mild withdrawal symptoms in the form of myalgia and bone pain. These symptoms occurred within the first month of discontinuation and were managed with occasional acetaminophen. Despite being scheduled for 3‐monthly follow‐ups and molecular assessments after discontinuing imatinib, 9 out of 45 patients (20%) had issues with compliance to their follow‐up visits. The median (range) delay in follow‐up was 36 (22–89) days in these nine patients.

Of the 12 patients with prior dual‐energy x‐ray absorptiometry (DXA) scans while on imatinib, improvement in bone mineral density (BMD) scores was observed in seven patients (58.3%) after 2 years of imatinib discontinuation (Figure S5).

## 6 Discussion

The incidence of CML is 10 times lower in children than that of adults [21] and existing treatment recommendations for treatment of pCML are mostly derived from adult guidelines. TFR has currently become one of the standard treatment goals in adults with CML following the success of the landmark discontinuation studies [7, 8]. Pediatric TFR studies are far and few, necessitating the need for more studies before TFR can be pronounced as one of the goals of therapy in pCML.

Initial studies on TFR in children showed inferior results compared to adult studies while the more recent ones have comparable outcomes as elaborated in Table 1 [9, 10, 11, 12, 13]. The definition of molecular relapse and the criteria to restart imatinib have a direct impact on the TFR rate [22]. Previously, any detectable BCR::ABL1 transcripts or loss of DMR would prompt a restart of imatinib therapy. Standard definitions for molecular relapse, that is, loss of MMR in contemporary trials have likely improved the TFR rate in both children and adults [23]. Based on the recent NCCN guidelines for adults we have taken the loss in MMR as the standard cut‐off to restart imatinib. We also chose to adapt the NCCN criteria for discontinuation over the ELN criteria, as the latter is more stringent, allowing a trial of TFR for patients only beyond 5 years of TKI treatment. Given the promising role of stopping TKIs early in children to mitigate the duration of exposure to TKI, which is often associated with several long‐term toxicities [3, 4, 5, 6], we chose 3 years on TKI with 2 years in DMR as the eligibility criteria in our patients.

**TABLE 1:** Pediatric data on discontinuation of TKI in CML‐CP.

Variables	International Registry of Childhood CML (2021) (10)	CML‐pediatric II study (2018) (13)	STOP IMAPED study (2019) (9)	Japanese study (2022) (11)	Study from India (2023) (12)	Present study (2024)
Total number of patients	581	140	470	152	—	186
Study	Retrospective	Retrospective	Retrospective	Prospective	Retrospective	Prospective
Criteria for stopping	MR4 for ≥ 2 years	≤ MR4.5 for a minimum of 12 months	—	Duration of TKI > 3 years MR4.0 for ≥ 2 years.	Duration of TKI > 5 years Undetectable BCR::ABL1 transcripts	Duration of TKI > 3 years MR4.0 for ≥ 2 years.
Number of patients in which TFR is attempted	18 (3%)	7 (5%)	14 (3%)	22 (14.5%)	11	45 (24.2%)
Median age at stopping of TKI	16 years (range, 9–24)	—	14 years (range, 9–23)	16 (5–26) years	—	20 (6–32)
Proportion of patients who successfully stopped TKI	56% (95% CI 33%–79%)	2/7 = 29%	4/14 = 28%	50% (90% CI: 32%–66%) at 12 months	8 (72%)	70% (95% CI 61%–88%) at 12 months 66% (95% CI 52%–84%) at 24 months
TKI used	Imatinib	Imatinib	Imatinib	Imatinib, 2G‐TKI and HSCT	Imatinib	Imatinib
Median duration of imatinib before discontinuation	73.2 months (range, 32–109)	44 months (range, 24–90)	64 months (range, 32–157)	100 (42–178) months	—	126 months (range, 46–262)
Duration of MR.4.0 before TKI discontinuation	46.2 months (range, 23.9–98.6)	—	—	53.5 (25–148) months	—	73.4 (46.8) months
Definition of molecular relapse	Loss of MMR or one log increase in transcript level	—	Reappearance of BCR:: ABL1 transcripts on 2 measurements, 2 to 4 weeks apart	Single loss of MMR (BCR::ABL1 transcripts > 0.1%)	Reappearance of BCR::ABL1 transcripts	Loss of MMR on two consecutive samples, repeated 4 weeks apart
Molecular relapse	7 out of 18	—	10 out of 14	CI MMR: 50% (90% CI: 31%–66%)	—	CI MMR loss at 12 months: 27% (95% CI 12%–39%) CI MMR loss at 24 months: 33% (95% CI 16%–46%)
Median time to molecular relapse	4.1 months (range, 1.9–6.4)	—	3 months (Range: 1–6)	90 (55–119) days	—	11 children within 6 months and 2 beyond 6 months
Median molecular RFS	Sustained DMR—23.9 months	—	4 months (95% CI 2.2–5.8 months)	—	—	—
Median time to re‐achieve MMR/ DMR	4.7 months (range 2.5–18)	Within 2 months	8.5 months (range 1–34).	Within 4 months	< 3 months	CI MMR regain at 12 months: 63% (95% CI 28%–84%) CI MMR regains at 24 months: 87% (95% CI 20%–98%)
Median follow‐up after TKI discontinuation	51 months (range, 6–100)	—	—	37 months (range: 24–41 months)	18 months (range: 3 months–5 years)	25 months (range: 6–42)

The relative lack of data, and consequently the absence of standard recommendations for TKI discontinuation in children creates fear and anxiety among physicians, parents, and children alike, making them hesitant to opt for stopping TKI therapy. It was reported that 9% of eligible patients in the International Registry of Childhood CML did not participate for these reasons [10]. One‐fourth of the total cohort of pCML patients in our hospital, and 93% of eligible patients, were enrolled in this study. This enrolment rate is promising and reassuring, significantly surpassing that of previously published pediatric discontinuation studies, as shown in Table 1.

This prospective study revealed a better TFR rate of around 70% at 12 months and 66% at 24 months with a median follow‐up of 25 months (range: 6–42 months). With the limitation of relatively short follow‐up, our cohort's TFR rate at 12 and 24 months was higher than that reported by previous pediatric studies. Though the median age and duration of imatinib treatment before the discontinuation of imatinib in our cohort were also more when compared to the other pediatric studies, they were not significantly associated with successful TFR in univariate analysis. The duration of DMR and time to attain DMR (*p* = 0.168) and MMR (*p* = 0.662) were also not significantly associated with TFR. Around 30% experienced loss of MMR, most of them within 6 months (85%) and were restarted on imatinib of which 77% (10 out of 13) regained MMR within a median of 3 months as observed in other pediatric studies (Table 1). The three patients who are yet to achieve MMR are on 3 monthly follow‐ups with serial BCR:: ABL1 transcript monitoring.

Changes in the host immune status have been shown to play a major role in predicting successful TFR in adult CML after TKI cessation [24, 25]. Persistence and subsequent resurgence of leukemic stem cells (LSC) is the postulated mechanism behind the recurrence of disease leading to failure of a trial of discontinuation and the immune system is thought to be an important tool to control/eradicate these LSCs [26, 27]. An increase in CD8+ cells or a decrease in Treg cells has been shown to enhance the antitumor response [28, 29]. In our cohort, we sequentially analyzed the absolute counts and relative proportions of T cells and NK cells after TKI discontinuation. A significant correlation was found between higher absolute Treg levels and lower CD3+ T‐cells at baseline and the occurrence of DF in our patients. However, unlike the findings by Ilander et al., we did not observe any correlation between NK cells and outcomes in the DF and TFR groups [25]. The only other factor that predicted successful TFR in pediatric CML, as reported by Shima et al., was imatinib levels immediately before discontinuation. A lower imatinib trough concentration is associated with a higher probability of TFR (*p* = 0.004), consistent with findings from the DESTINY trial, which demonstrated higher TFR rates in patients who maintained remission despite being on a reduced dose of imatinib [11, 30]. Therapeutic drug‐level monitoring of imatinib done previously in our patients did not correlate with treatment response and was therefore discontinued in our center.

Withdrawal symptoms of TKI, including bone pain and myalgia, were observed in about 9% of our patients and were well‐controlled with over‐the‐counter acetaminophen. Although pediatric studies have not commonly reported withdrawal symptoms, Satishkumar M et al. have noted dry skin, myalgia, and hair loss following TKI cessation [12].

Existing guidelines recommend assessing BCR::ABL1 transcripts every 4 weeks during the first year, every 2 months in the second year, and every 3 months thereafter [23]. Our patients come from various parts of the country, making monthly follow‐up visits to our hospital impractical. Additionally, outsourcing qPCR tests to local laboratories can be costly and unreliable. Therefore, we initially opted for a monitoring frequency of every 3 months. As an additional precaution, we advised patients to have monthly blood counts done locally for the first 3 months. In one instance, a patient's white cell count increased significantly within a month of discontinuation, prompting an early qPCR that revealed a high transcript level. We have revised our monitoring schedule based on our experience with a few patients losing response and showing elevated transcript levels by 3 months. Following modeling on the safe minimum frequency of molecular monitoring by Shanmuganathan et al. [31], the first qPCR is now conducted 2 months after discontinuation, followed by 3‐monthly tests as previously planned.

Adherence to follow‐up after TKI cessation is another challenge, especially in low and middle‐income countries. In our cohort, 20% of patients were non‐compliant with even the 3‐monthly BCR::ABL1 monitoring during the first year of imatinib cessation.

The alleviation of TKI‐related toxicities after discontinuation has been reported in children including potential improvements in academic performance and growth if TKI is discontinued before puberty [11]. In our study, BMD improved in around 58% of patients after 2 years of discontinuation. This reinforces the fact that children may benefit from TKI discontinuation, with subsequent improvement in long‐term off‐target TKI‐related toxicities, particularly in the sectors of neuro‐cognition, growth, and bone health. Our patients continue to be followed up for any impact of TFR on long‐term toxicities experienced by them [4].

## 7 Conclusions

We observed a high TFR rate in children with CML‐CP in our cohort, the modified and less frequent BCR::ABL1 transcript monitoring strategy after imatinib cessation was found to be pragmatic and reasonably safe. The link between absolute T‐reg levels and successful TFR highlights the immune system's crucial role in achieving and maintaining TFR, which could influence future discontinuation trials in children. For patients experiencing DF, resuming imatinib typically restored MMR within an acceptable timeframe in most of the patients. Furthermore, long‐term discontinuation was associated with improved bone health in some patients. Multi‐center prospective studies are essential to establish TKI discontinuation as the standard of care in pCML.

## Author Contributions

N.R.M. and S.B. designed the study. S.K., N.R.M., and G.C. wrote the manuscript. G.C. helped in immune cell characterization. N.R.M., S.K., J.A., and P.R. performed data analysis. J.A. helped in data collection. S.B., G.N., C.D., A.C., and S.S. helped in patient management. P.M., H.J., D.S., P.T., N.P., and P.G.S. contributed from the cytogenetics and hematopathology laboratories. All the authors reviewed the manuscript submitted for publication.

## Ethics Statement

The prospective study was approved by the institutional review board and ethical committee clearance was obtained (ECR/414/Inst/MH/2013/RR‐19‐Study P. No. 3435).

## Consent

Informed consent was obtained from the patients/caregivers in our study.

## Conflicts of Interest

The authors declare no conflicts of interest.

## Supporting information


**Data S1.** Supporting Information.

## Data Availability

The data that support the findings of this study are available from the corresponding author upon reasonable request.
