# Supplementary material for: Treatment‐Free Remissions in Children With Chronic Myeloid Leukemia (CML): A Prospective Study From the Tata Memorial Hospital (TMH) Pediatric CML (pCML) Cohort
Source: Am J Hematol. 2024 Nov 20;100(2):210–7. doi: 10.1002/ajh.27528 (PMC11705202; doi:10.1002/ajh.27528)
Supplement: Supplementary file 1 — Data S1. Supporting Information. [file AJH-100-210-s001.docx]

**Supplementary Figure 1** demonstrates the template-based, sequential gating strategy used to identify various immune cells (CD4+ and CD8+ T cells, γδ-T cells, Tregs, NK cells, early, effector and terminal NK cells, and CD159a+ exhausted NK cells). This gating strategy is modified from our previously published protocol (15).


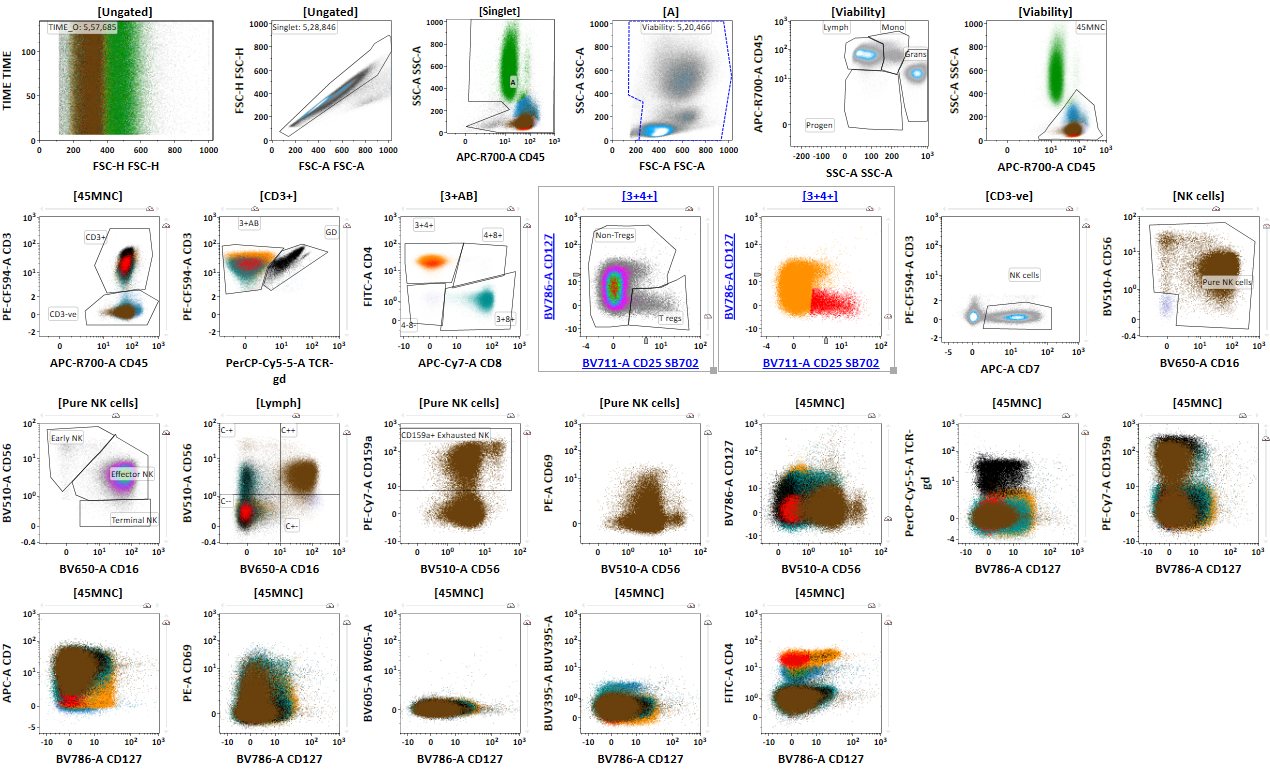


**Supplementary Figure 2:** Pattern of change in absolute levels of Tregs after 3 months of discontinuation. The red line denotes the ROC determined cut-off of 9.14/μL.


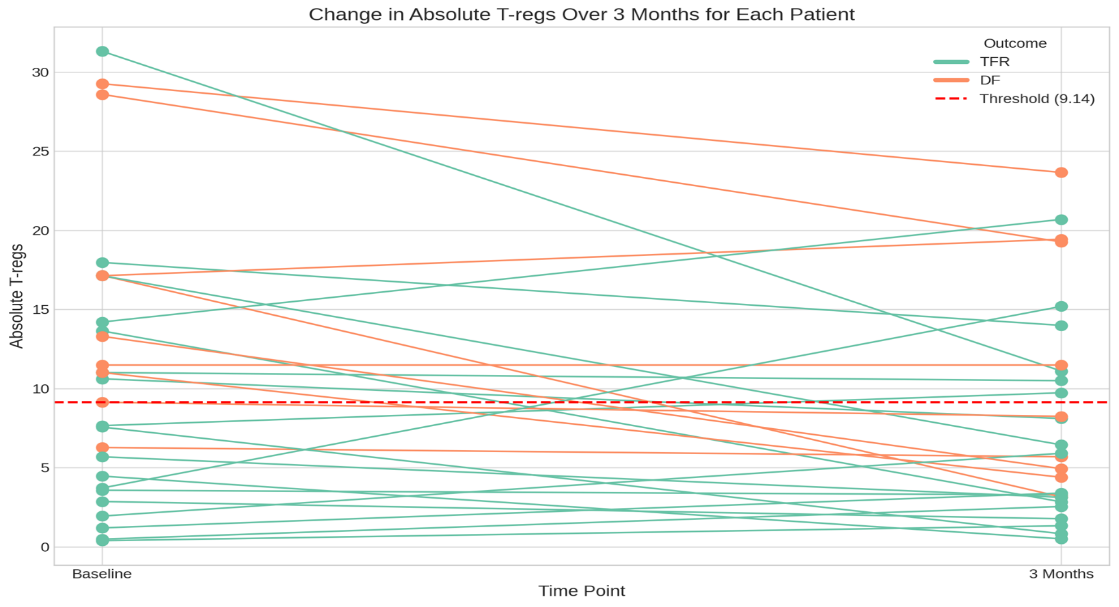


**Supplementary Figure 3:** Pattern of change in proportion and absolute levels of various immune cells after 3 months of discontinuation.


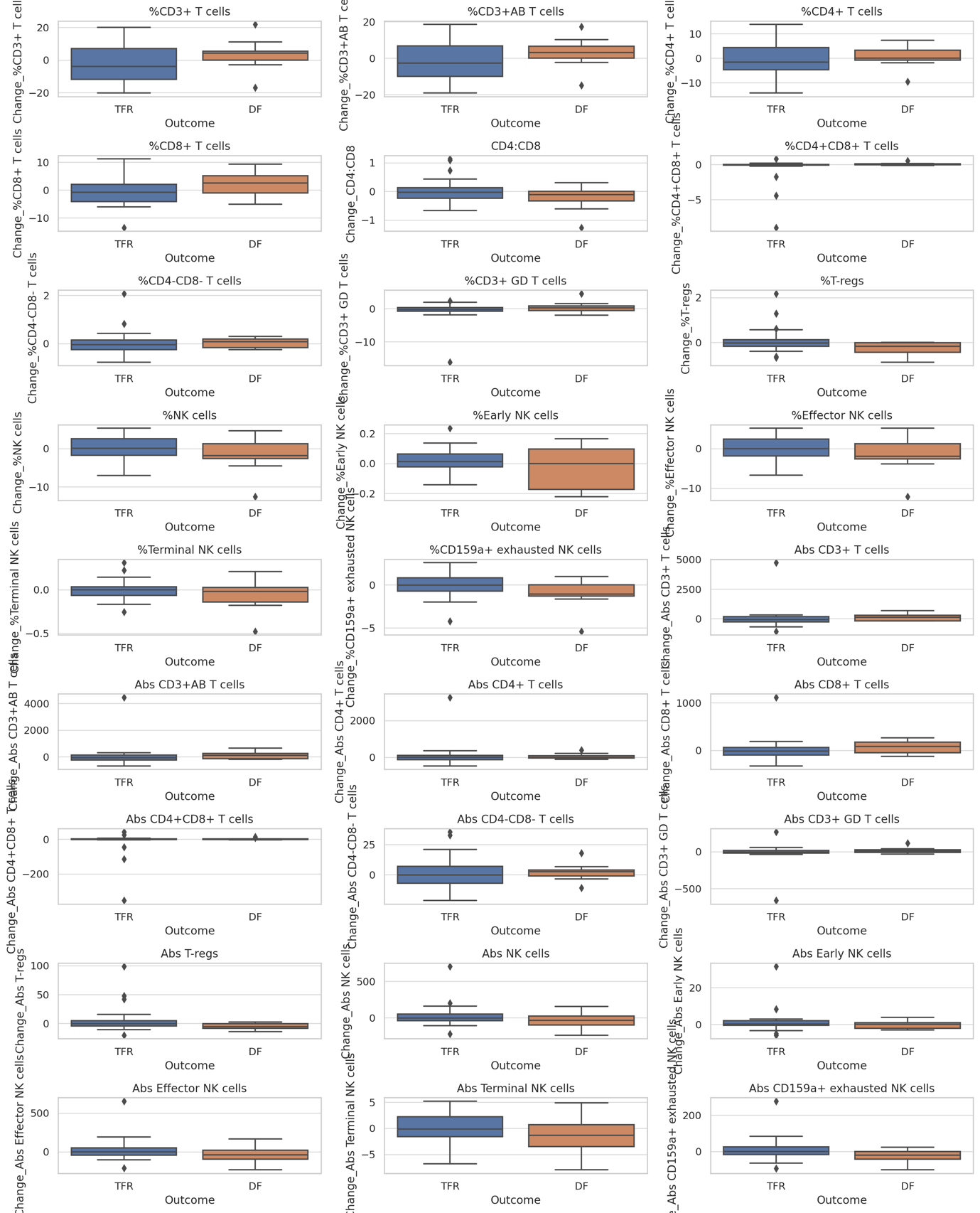


**Supplementary Figure 4** shows the loss of DMR in 10 patients who remained on TFR.


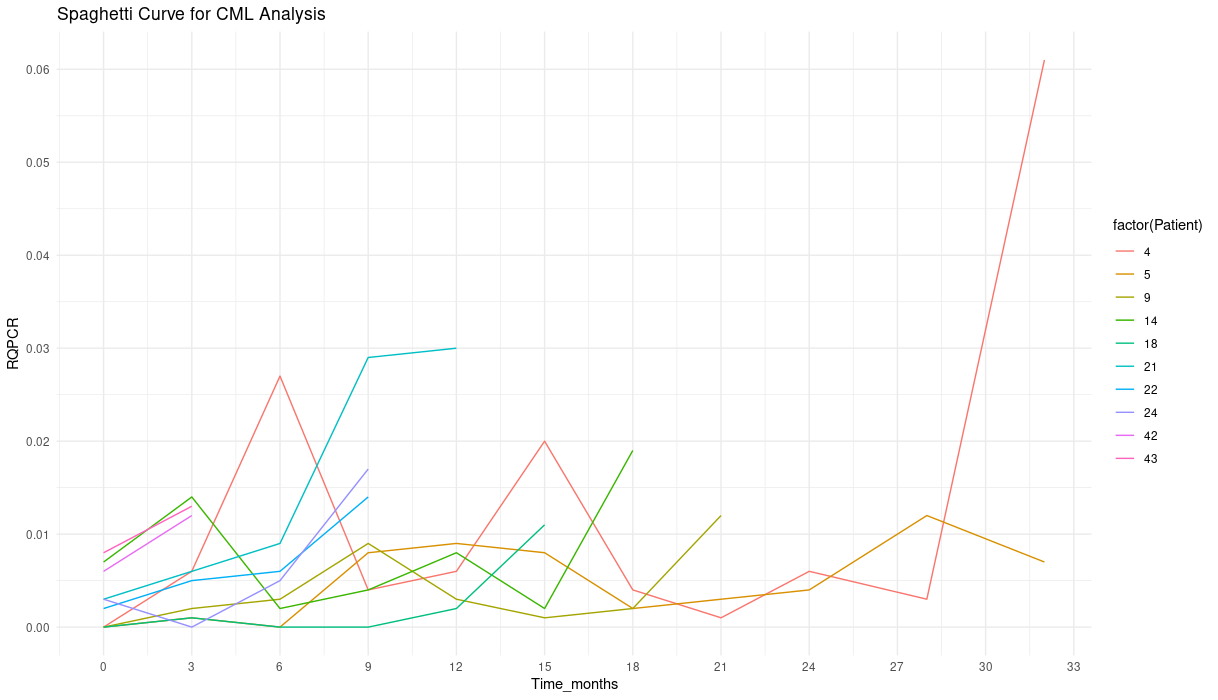


**Supplementary Figure 5** shows improvement in BMD scores (n=7) after 2 years of TFR

**Supplementary Table 1: 12-color antibody panel used for immune cell characterization**

|  | BUV 737 | BUV 395 | BV786 | BV711 | BV650 | BV605 | BV510 | BV421 | FITC | PE | ECD | PC5.5 | PC7 | APC | APC-A700 | APC – A750 |
| --- | --- | --- | --- | --- | --- | --- | --- | --- | --- | --- | --- | --- | --- | --- | --- | --- |
| Antibody | X | X | **CD127** | **CD25** | **CD16** | X | **CD56** | X | **CD4** | **CD69** | **CD3** | **TCRGD** | **CD159a** | **CD7** | **CD45** | **CD8** |
| Clone |  |  | HIL-7R-M21 | BC96 | B73.1 |  | NCAM16.2 |  | 13B8.2 | 84H10 | UCHT1 | IMMU 510 | Z199 | 8H8.1 | J.33 | B9.11 |
| Vol |  |  | 0.25 ml | 0.5 ml | 0.25 ml |  | 0.25 ml |  | 0.5 ml | 2 ml | 1 ml | 0.5 ml | 0.5 ml | 0.5 ml | 1 ml | 0.5 ml |
